# Performance of Targeted Library Preparation Solutions for SARS-CoV-2 Whole Genome Analysis

**DOI:** 10.3390/diagnostics10100769

**Published:** 2020-09-29

**Authors:** Petr Klempt, Petr Brož, Martin Kašný, Adam Novotný, Kateřina Kvapilová, Petr Kvapil

**Affiliations:** 1 Institute of Applied Biotechnologies, Služeb 3056/4, 108 00 Prague, Czech Republic; broz@iabio.eu (P.B.); kasny@iabio.eu (M.K.); novotny@iabio.eu (A.N.); kvapilova@iabio.eu (K.K.); kvapil@iabio.eu (P.K.); 2 Department of Biology and Medical Genetics, Second Faculty of Medicine, Charles University and Motol University Hospital, V Úvalu 84, 150 06 Prague, Czech Republic; 3 Faculty of Science, Charles University, Albertov 6, 128 00 Prague, Czech Republic

**Keywords:** SARS-CoV-2, NGS, genome variant, Illumina, Paragon, Twist

## Abstract

Single next-generation sequencing (NGS) proved to be an important tool for monitoring the SARS-CoV-2 outbreak at the global level Until today, thousands of SARS-CoV-2 genome sequences have been published at GISAID (Global Initiative on Sharing All Influenza Data) but only a portion are suitable for reliable variant analysis. Here we report on the comparison of three commercially available NGS library preparation kits. We discuss advantages and limitations from the perspective of required input sample quality and data quality for advanced SARS-CoV-2 genome analysis.

## 1. Introduction

The global spread of a new type of coronavirus, SARS-CoV-2, causing the respiratory disease COVID-19 [1,2,3,4] mobilized both the public and private sector and resulted in a rapid development of solutions focused on SARS-CoV-2 detection and analysis. Next to a number of solutions utilizing the advantages of RT-qPCR techniques for SARS-CoV-2 detection [5,6], the next-generation sequencing (NGS)-based protocols allows the analysis of SARS-CoV-2 genome evolution and variability and the monitoring of its spread within the global population (Nextstrain; https://nextstrain.org) [7,8]. These knowledges address the need to elucidate its genomic characteristics (GISAID; https://www.gisaid.org) in order to ensure the efficiency of RT-qPCR testing, assess its transmission through clonal events, and develop a reliable vaccination protocols future therapies, especially considering the fact that RNA viruses are prone to accumulate variants in its genome in a relatively short timeline, which in the case of SARS-CoV-2 is also related to its capacity to proofread and remove mismatched nucleotides during genome replication and transcription [9,10,11,12]. In general, the various types of kits stem from two dominant approaches—amplicon and hybridization capture. In general, both approaches show significant advantages and disadvantages in the term of workflow, sample input and NGS library output requirements [13,14]. Amplicon-based methods are in general based on a simpler workflow and require smaller amounts of input DNA, but they are typically limited to small panels (a few hundred amplicons), and tend to have a high PCR background and lower uniformity of coverage [13]. The capture-based approaches are more useful for targeting large regions, such as small (viral) genomes and whole exomes, and sequencing data shows better uniformity of coverage. Concerning the workflow, the capture-based based approaches are more laborious and require relatively large inputs of nucleic acids [13]. Recently, several companies have developed SARS-CoV-2 targeted NGS library preparation kits intended for use on Illumina NGS platforms. In this study we report performance analysis of three commercially available library preparation kits, utilizing both amplicon (Paragon Genomics, Hayward, CA, USA) as well as hybridization capture (Illumina, San Diego, CA, USA, Twist Bioscience, San Francisco, CA, USA) approaches (see the links in Material and Methods). We predefined a number of evaluation criteria to address the most important wet-lab (such as sample quality and input requirements, laboriousness of workflow including a time, quality of NGS library, costs) as well as data analysis steps (such as % of Alligned Bases, % of Duplicates, % of Mapped Reads, Mean Target Coverage) with a main goal—to achieve high quality genomic data for subsequent reliable variant analysis of SARS-CoV-2 genome. In total 55 RNA isolates of nasopharyngeal swabs (NS) or bronchoalveolar lavage fluid (BLF) from five different laboratories located in Czech Republic were analysed. In order to validate our workflow (wet lab as well as bioinformatics pipeline) we decided to engage two synthetic SARS-CoV-2 positive controls (Twist Bioscience, San Francisco, CA, USA), corresponding to different variants of the virus genome (MT007544.1 and MN908947.3), combined in four different dilutions, resulting in 3, 10, 100 and 1000 copies used for NGS library preparation by each of three approaches as well as artificial negative control (total human RNA, Takara, Takara, Saint-Germain-en-Laye, France).

## 2. Materials and Methods

### 2.1. Samples

All samples originate from patients positively diagnosed with COVID-19. Sampling took place in five Czech hospitals—two in Prague, two in Brno, one in Plzeň. In total, we have received 55 samples of nasopharyngeal swabs from positive cases. Isolation of total nucleic acid content from nasopharyngeal swabs was performed by QIASymphony Virus/Pathogen Mini kit and/or QIAamp Viral RNA Mini kit K (Qiagen, Germantown, MD, USA) respectively (depending on their availability) according to manufacturer’s instructions to obtain isolates. Viral content of the isolates was subsequently quantified by RT-qPCR (Direct SARS-CoV-2 RT PCR kit, Institute of Applied Biotechnologies, Prague, Czech Republic) in order to evaluate the copy number of SARS-CoV-2, based on WHO-recommended protocol (www.who.org). In direct SARS-CoV-2 RT PCR assay we used the novel coronavirus sequences reported by the United States Centers for Disease Control targeting two genetic sequences of the viral nucleocapsid (*N1*, *N2* genes) and human RNase P (*RP* gene) as an internal control [15].

### 2.2. Controls Generation

Negative RNA controls prepared from 5ng (nc) human breast tumor RNA (HBT) (Takara Bio; Saint-Germain-en-Laye, France). Positive controls (pcs) were prepared by spiking 5 ng of HBT with 2 synthetic SARS-CoV-2 RNA controls (SARS-CoV-2 RNA control 1, SARS-CoV-2 RNA control 2; Twist Bioscience; USA) in order to reach 3 (pc1), 10 (pc2), 100 (pc3), 1000 (pc4) copies of both synthetic genomes within one control. The prepared controls were qualified by RT-qPCR (Ct values).

### 2.3. Calculation of Ct for Samples and Controls

The number of virus copies in samples was calculated according to Ct values measured by adoption of direct SARS-CoV-2 RT PCR assay for synthetic RNA positive controls diluted to defined viral copy concentration. The direct SARS-CoV-2 RT PCR results were validated with IVD CE certified kit UltraGene Combo2Screen SARS-CoV-2 Assay (ABL SA Group, Luxembourg) on RNA isolated from nasopharyngeal swab samples using a viral RNA isolation kit from cell-free fluids (NucleoSpin RNA Virus, Macherey-Nagel, Duren, Germany).

### 2.4. NGS Library Preparation

Overall, NGS libraries were prepared from 55 isolates in sets specific for each library preparation solution. Thirteen samples were prepared using all three kits.

#### 2.4.1. Library Preparation—1st Attempt NEB+TWIST (NEB+TWIST1)

The library was prepared from a set of 24 isolates (Ct range: 11.29–31.96; see Supplementary Table S1) and 4 positive (pc1, pc2, pc3, pc4) and 2 negative (nc) controls using NEBNext Ultra II Directional RNA Library Prep Kit for Illumina (New England Biolabs, Ipswich, MA, USA) following manufacturer’s protocol (Protocol NEBNext Ultra II Directional RNA Library Prep Kit for Illumina (NEB #E7760, #E7765; https://international.neb.com/-/media/nebus/files/manuals/manuale7760_e7765.pdf).

Three enrichment plexes (10 samples each, 1500 ng in total per plex) were prepared by pooling 150 ng or maximal available amount of each sample (if available) as described in Supplementary Table S1. Plexes were subsequently enriched using the Twist SARS-CoV-2 Research Panel kit (Twist Bioscience; https://www.twistbioscience.com/sites/default/files/resources/2020-07/ProductSheet_NGS_SARS-CoV-2_Panel_14JUL20_Rev1.2.pdf), following the manufacturer’s protocol (Twist Target Enrichment Protocol; https://www.twistbioscience.com/sites/default/files/resources/2020-07/Protocol_NGS_EnzymaticLibraryPrepCDI_13JUL20_Rev1.1.pdf, https://www.twistbioscience.com/sites/default/files/resources/2019-11/Protocol_NGS_HybridizationTE_31OCT19_Rev1.pdf). Enriched plexes were equally pooled based on evaluation by Qubit 2.0 and Bioanalyzer 2100 and sequenced on the MiSeq platform using MiSeq Reagent Kit v3 (600 cycle) (Illumina, San Diego, CA, USA).

#### 2.4.2. Library Preparation—2nd Attempt NEB+TWIST (NEB+TWIST2)

The second library preparation followed the same procedure as the first experiment, with exception of utilizing the knowledge of Ct values of samples (Ct 13,1–19). 17 NGS libraries were combined into 2 plexes (6-plex and 11-plex) based on Ct values in order to minimize the range span (see Supplementary Table S1).

Enriched plexed were evaluated by Qubit 2.0 and Bioanalyzer 2100, pooled equally (with regard to the number of samples in individual plexes) and sequenced using MiSeq platform using Reagent Kit v2 (500 cycle) (Illumina, San Diego, CA, USA).

#### 2.4.3. Library Preparation—Illumina

A set of 35 isolates (Ct values 11.29–40, Supplementary Table S1)—4 positive (pc1, pc2, pc3, pc4) and 1 negative (nc2) controls—was transcribed into ds cDNA using NEBNext^®^ RNA First Strand Synthesis Module and NEBNext^®^ Ultra™ II Directional RNA Second Strand Synthesis Module (New England Biolabs; Ipswich, MA, USA) following manufacturer’s protocol.

Libraries were prepared using Nextera Flex for Enrichment (pre-enrichment part of manufacturer’s guide; https://emea.support.illumina.com/content/dam/illumina-support/documents/documentation/chemistry_documentation/illumina_prep/illumina-dna-prep-with-enrichment-reference-1000000048041-05.pdf). Next, libraries were combined within 8 plexes by 5 samples each based on Ct values (see Supplementary Table S1) and enriched using the Respiratory Virus Oligo Panel (Illumina, San Diego, CA, USA, following the manufacturer’s protocol (https://www.illumina.com/content/dam/illumina-marketing/documents/products/appnotes/coronavirus-enrichment-product-list-1270-2020-004.pdf).

Enriched plexes were equally pooled based on evaluation by Qubit 2.0 and Bioanalyzer 2100 and sequenced on the MiSeq platform using MiSeq Reagent Kit v3 (600 cycle) (Illumina, San Diego, CA, USA).

#### 2.4.4. Library Preparation—Paragon

For the NGS library construction with the amplicon approach, the identical set of samples as the one used in the Illumina approach was used. Briefly, 35 isolates, four positive and one negative controls were prepared using the CleanPlex^®^ SARS-CoV-2 Research and Surveillance Panel (Paragon Genomics, San Francisco, CA, USA), following the manufacturer’s protocol (https://www.paragongenomics.com/wp-content/uploads/2016/12/UG1001-06-CleanPlex-NGS-Panel-User-Guide.pdf) using a variant of two-pooled multiplex PCR reactions (see manufacturer’s protocol). From a total of 40 preparations, only 21 libraries were finally successfully prepared (evaluated from Bioanalyzer traces as recommended by the guide) in sufficient quality for sequencing.

21 purified libraries were pooled (due to low concentration of some libraries we were not able to pool each of the libraries equally, see Supplementary Table S1) and sequenced on the MiSeq platform using Reagent Kit v3 (600 cycle) (Illumina).

### 2.5. Sequencing

Libraries from each preparation were pooled based on the quality control evaluation. Further, libraries were diluted and denatured according to the MiSeq Denature and Dilute Guide (February 2019, v10 version). Final loading concentration, as well as sequencing configuration specified in Supplementary Table S2.

### 2.6. Reference Mapping and Bioinformatic Analysis

FastQC v. 0.11.8 [16] was used to control sequence quality prior to trimming. Low quality, short reads and adapters were trimmed by fastp 0.20.0 [17]. The remaining reads were aligned to reference genome NM9088947.3 (which is identical to NC_045512) using bowtie2 [18] with default sensitive (--sensitive D 20 -R 3 -N 0 -L 20 -i S,1,0.50) parameters. Flagging duplicate reads (in enrichment libraries) and calculation of read depth were done by Picard [19]. Sequencing and bioinformatics workflow were validated by identification of variants present in the synthetic control samples (pc4) based on MT007544.1 and NM9088947.3 reference genome (Table 1). Variant calling was performed using the Freebayes algorithm with default parameters. The median coverage in all coding sequences (CDS) of SARS-CoV-2 gene regions (ORF1a, ORF1ab, S, ORF3a, E, M, ORF6, ORF7a, ORF7b, ORF8, N, ORF1) was calculated.

## 3. Results and Discussion

### 3.1. NEB+Twist Workflow

**Figure 1:**
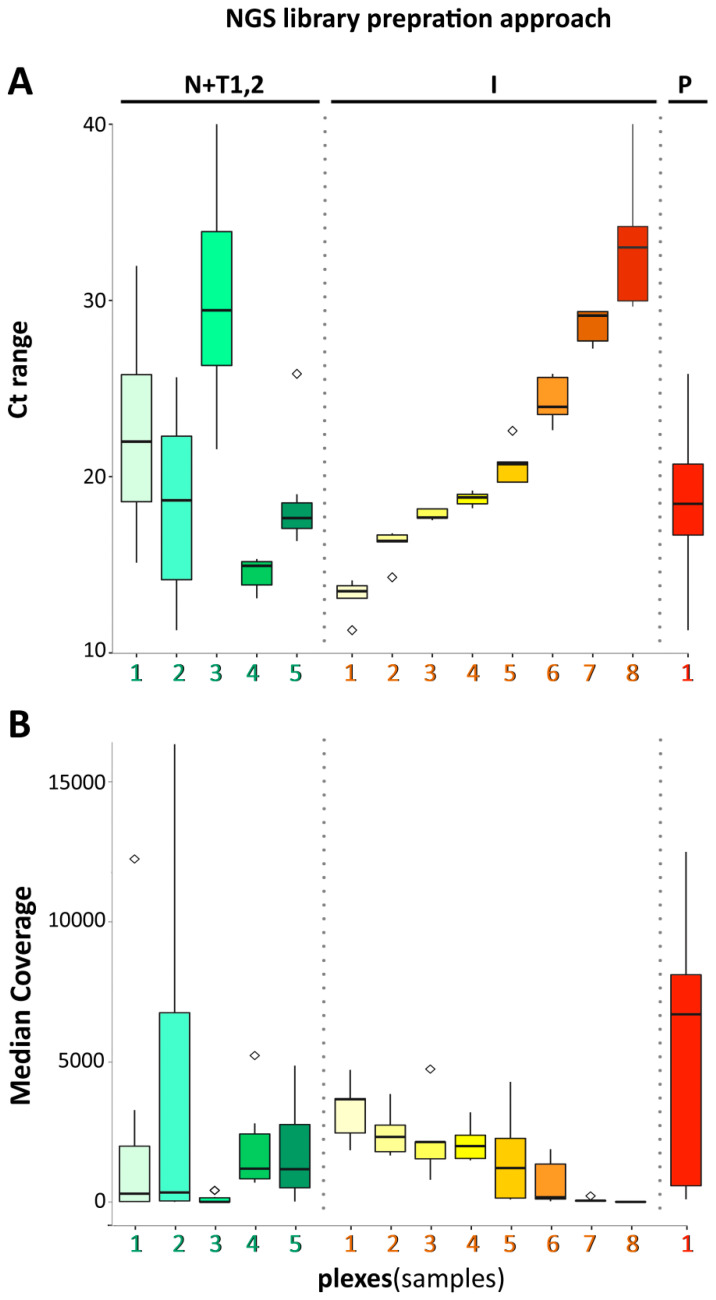
(**A**) Distribution of Ct values within the particular plexes of NEB+TWIST1,2; N+T1,2 (NEB+TWIST1 plex 1–3, NEB+TWIST2 plex 2–3), ILLUMINA; I (plex 1–8) and PARAGON; P (all the samples) approach. (**B**) Distribution of median coverage of reads which were mapped to target regions within the particular plexes of NEB+TWIST1,2; N+T1,2 (NEB+TWIST1 plex 1–3, NEB+TWIST2 plex 2–3), ILLUMINA; I (plex 1–8) and PARAGON; P (all the samples) approach. Box and Whiskers plots represent the median (horizontal line), 25th and 75th percentiles (boxes), maximum and minimum (whiskers) and outliers (◊).

During the first attempt, thirty libraries (including 4 positive and 2 negative controls) were prepared in three plexes (10 samples each, see Supplementary Table S1) employing NEBNext^®^ Ultra™ II Directional RNA Library Prep Kit for Illumina (New England Biolabs) followed by capture-based workflow utilizing the Twist SARS-CoV-2 Research Panel (Twist Bioscience). Bioinformatic analysis of sequenced data showed high variability of data quality among samples within the experiment (e.g., mean coverage and total number of reads) as well as within each of plexes (see Figure 1 and Supplementary Table S1). Contrary, even distribution of the total reads over three plexes (19, 23.6 and 20.1 M PE reads) confirmed the pooling efficiency. Varying sample performance within plex (Supplementary Table S1, STD 4.65 M, 3.93 and 4.63 M PE reads per plex 1, 2 and 3) requested re-evaluation of provided Ct measurement metrics, and led us to standardize Ct value of all samples by in-house RT-qPCR sample rating assay (IAB; see Materials and Methods), instead of using of data provided by external laboratories. This solution was subsequently proved by the second NEB+Twist experiment, which resulted in more even coverage distribution among 17 libraries (one positive control) prepared in two plexes (STD within plexes 1.05 and 1.78 M). These data indicate that plexes composed of samples with similar Ct value show higher uniformity of coverage within the plex compared to plexes prepared without respect to re-evaluated Ct value (Figure 1).

### 3.2. Illumina Workflow

Subsequently, knowing the importance of Ct evaluation for sample QC, 40 NGS libraries were prepared by the Illumina protocol, utilizing the Respiratory Virus Oligo Panel in combination with Nextera Flex for Enrichment (NEB solutions was used for first and second strand cDNA generation, see Materials and Methods).

Forty libraries (including 4 positive and 1 negative controls) were enriched in 8 plexes with the goal to minimize the Ct sample range per plex to a difference of maximum 3 where possible (exception in plex 8, see Supplementary Table S1). Generally, lowering the number of samples within each plex of capture-based workflows (can go down to single-plex) raises the chance of equal distribution of sequencing capacity amongst samples (see Figure 1 and Supplementary Table S1), however also dramatically raises the price per sample as well as hands-on time.

Compare to NEB+TWIST the capture-based Illumina approach data showed in average a lower percentage of mapped reads (Supplementary Table S1), this consequence could be related to the lower specificity of Respiratory Oligo Viral Panel designed next to the SARS-CoV-2 also for other pathogens (see https://www.illumina.com/content/dam/illumina-marketing/documents/products/appnotes/ngs-enrichment-coronavirus-app-note-1270-2020-002.pdf).

### 3.3. Paragon Workflow

Unlike capture-based protocols, Paragon’s amplicon-based workflow keeps samples separated during the whole workflow (Supplementary Table S1). Therefore, it is potentially possible to directly evaluate the success of prepared targeted SARS-CoV-2 NGS library (see Materials and Methods). In our hands, Paragon protocol resulted in 20 (plus 1 control) successfully generated libraries out of 40 samples in total. Since the same set of samples was also used for the Illumina workflow where it resulted in 35 sequenced genomes (plus 4 controls), the further optimization of the Paragon protocol would be needed. Paragon NGS library preparation workflow was proved to be the fastest protocol out of three mentioned solutions (see comparison below).

### 3.4. Data Analysis

The main quality control parameter is the fraction of all target bases which achieved at least 20× coverage or greater. Following this arbitrary limit, sample analysis was considered as successful in case it reached a minimal 50% proportion of targeted bases with coverage ≥20×. Out of 95 sequenced libraries eighty-six libraries passed this limit; 16 sample libraries and 2 control libraries of 1st attempt NEB+Twist workflow (out of 30), 16 sample libraries of 2nd attempt NEB+Twist workflow (out of 17), 30 sample and 2 control libraries of Illumina workflow (out of 35) and all 21 sequenced Paragon workflow libraries including one control library (Supplementary Table S1). The impact of Ct on this parameter is especially apparent from the comparison of the first and second approach using the NEB+Twist workflow (see Figure 1 and Supplementary Table S1). Also, the viral load in a sample (corresponding to sample Ct value ≤ 23.29 or positive control value ≤ 25.84) showed to be the limiting factor in case of each workflow, samples with higher Ct value resulted in either poor genome coverage (NEB+Twist workflow and Illumina workflow) or in absence of expected library preparation product (Paragon workflow).

Considering the time-consumption of each library preparation approach, the fastest was amplicon-based Paragon workflow with hands on time (HOT) 4 12min and 6 h 30 min instrument time (INT), followed by Illumina (6 h 6 min HOT and 23 h 24 min INT) and NEB+Twist workflow (6 h 42 min HOT and 26 h 54 min INT) (Figure S1). This is in concordance with the expectations based on the manufacturer’s manual as well as the general principle of each workflow. It should be noted that Illumina workflow allows faster hybridization time (down to 90 min; we used 5 h long incubation) which reduces the time difference between enrichment and amplicon-based approaches.

**Figure 2:**
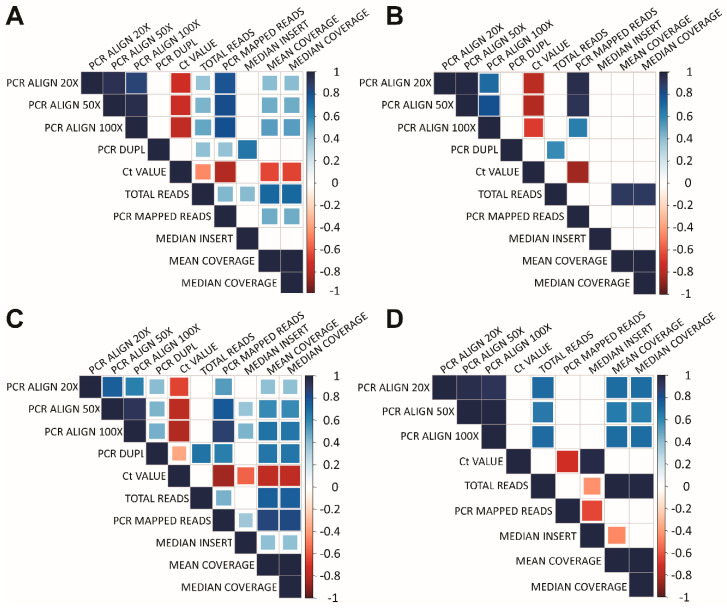
Mutual correlation of selected categories of metrics related to (**A**), NEB+TWIST1 (plex 1–3); (**B**), NEB+TWIST2 (plex 2–3); (**C**), ILLUMINA (plex 1–8); and (**D**) PARAGON (no plex) approach. We can observe a significant negative correlation between Ct value and % of mapped reads to the reference genome. Lower Ct number caused higher number of % mapped reads and therefore results in better coverage. PRC ALIGN 20×, the percentage of bases that were aligned to the reference sequence at least 20×; PRC ALIGN 50×, the percentage of bases that were aligned to the reference sequence at least 50×; PRC ALIGN 100×, the percentage of bases that were aligned to the reference sequence at least 100×; PRC DUPL, percentage of duplicate sequences (not generated in the case of PARAGON approach); Ct VALUE, reflecting the viral load; TOTAL READS, total number of reads which were yielded by sequencing on MiSeq (Illumina); PCR MAPPED READS, total number of reads which were mapped to target regions; PERC_MAPPED, percentage of mapped reads which were mapped to target regions; MEDIAN INSERT, median value of insert length; MEDIAN COVERAGE, median coverage of reads which were mapped to target regions; MEAN COVERAGE, mean coverage of reads which were mapped to target regions.

**Figure 3:**
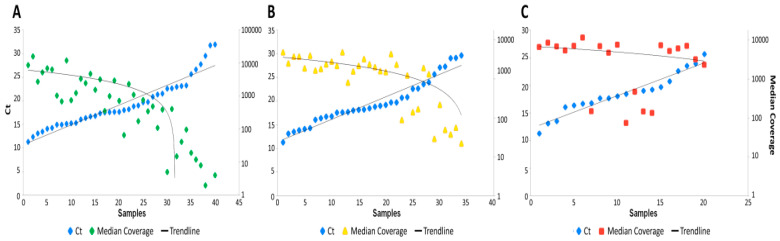
Correlation of Ct (reflecting the viral load) to median coverage of reads which were mapped to target regions. Sequenced samples without controls: NEB+TWIST1,2; (**A**) 40 samples, Illumina; (**B**) 35 and Paragon; (**C**) 20 (19 prepared amplicon libraries, including 4 controls, were excluded from sequencing—did not meet the QC limit). (In a graph NEB+TWIST1,2 are presented together with samples of both independent preparations). Min/max Ct values: NEB+TWIST1,2 (11.29–32.96), Illumina (11.29–29.98) and Paragon (11.29–25.63). Min/max median coverage values: NEB+TWIST1,2 (0–16015), Illumina (9–4796) and Paragon (73–11415).

From the perspective of quality of generated sequencing data, the Ct value showed to be the major predictor of sample outcome in regards to the goal of reaching 20× coverage obtained on a particular sample, as well as success of library preparation, regardless of type of workflow. Since Ct reflects the actual viral load, it has negative correlation with almost all monitored parameters (Figure 2 and Figure 3).

**Figure 4:**
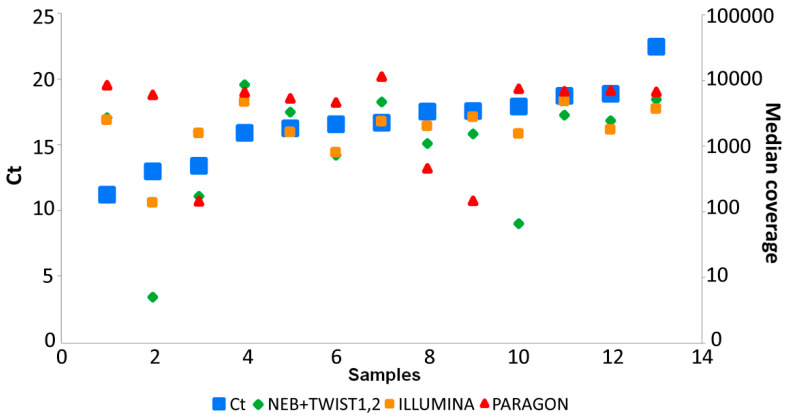
Correlation of Ct values (reflecting the viral load) to median coverage of reads for 13 samples processed by all three approaches (NEB+TWIST1,2, both; ILLUMINA; PARAGON). Min/max Ct values 11.29–22.6.

In respect to the aim of obtaining >20× coverage, based on presented data, the recommended samples are those with Ct value among 11–23. Samples within this range required a lower number of total reads and higher ratio of mapped reads (Supplementary Table S1) compared to those with Ct above 23. In the case of data recorded on the set of 13 samples which were prepared by all three approaches the negative correlation of median coverage to Ct values did not show such significant trend because all the samples were in the optimal Ct values range 11.29–22.6 (Supplementary Table S1, Figure 4). Nevertheless our data demonstrate that even samples with Ct values ≥ 23 can be successfully processed, however will require substantially higher sequencing capacity and tend to be more prone to dropouts.

Enrichment based approaches required a higher number of reads (min. 109 624 PE reads, IAB20_006_34 sample, see Supplementary Table S1) to reach the optimal coverage limit than amplicon approach (75 684 PE reads, IAB20_006_35), which is in concordance to expectation that amplicon-based approach have generally better on-target rate compared to capture-based approach [13]. On the other hand, the evenness of coverage was better in both capture-based protocols compared to amplicon-based, as shown by Supplementary Table S1 and Figure 3, which is also in concordance with previously published results [18]. It should be noted that Illumina´s panel also targets several other viral genome sequences (21 viruses in total; performance not evaluated) as well as human targets serving as positive control, which is reflected by increased panel size as well as sequencing requirements (see the link in Materials and Methods).

In all CDS regions the read depth is higher compare to median coverage across whole genome (see Reads of Depth for CDS in Supplementary Table S1). We can observe 0 read depth coverage in all CDS region of negative control samples (nc). There are also dropouts in NEB+TWIST1 method in samples IAB20_006_03 and IAB20_006_13. This gap corresponds to low mapping rate (% mapped reads) to the reference genome (Supplementary Table S1). Except for the mentioned dropouts, we can observe relatively good read depth across whole CDS regions.

Whole experiment was done during the acute phase of the coronavirus crisis, using routinely-generated clinical isolates. These samples represent certain constraints for successful NGS library preparation and/or data processing, due to limited concentration (often below detection threshold of Qubit HS Assay), variable quality and viral load and limited volume. Therefore, it was not possible to apply all three methods on the whole set of samples, and 13 samples were prepared using all three methods. We present relevant data acquired in this set of samples in Figure 4 and Supplementary Table S1 (highlighted in red), however due to the observation that performance of given sample is also affected by sample composition (number and quality) of enrichment plexes (the effect of Ct), we suppose that the performance in regards to solution used for library preparation is rather relative.

In regards to data analysis of obtained viral genome sequences, we decided to validate reference-mapping-based pipeline using the knowledge of presence of variants within synthetic Twist control (3–1000 copies per reaction volume, see Materials and Methods). Positive control was prepared from two synthetic controls representing two genome variants: MN9088947.3 and MT007544.1 (Twist Bioscience). Thus, 2 genome variants combined in positive control differed by 4 sites: 3 SNPs (19065T > C, 22303T > G, 26144G > T) and one deletion (29749 ACGATCGAGTG > A). We filtered out the mapped reads of low quality (MQ < 20), bases with lower quality (BQ < 25), supplementary and secondary alignment, PCR duplicates (except Paragon which is an amplicon-based approach), read depth (DP > 20×). For high quality variant calling, Freebayes algorithm (default parameters) was used [20]. For the Paragon data the variant calling was performed after trimming out the primer sequences to minimize false positive results.

**Figure 5:**
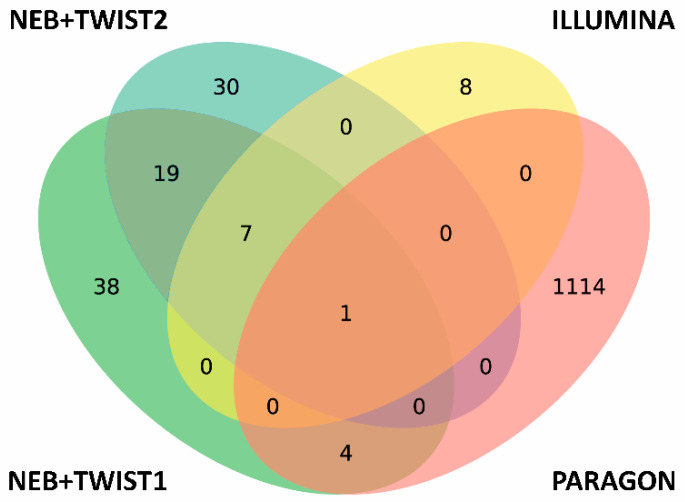
Venn diagram showing the distribution of variants (SNPs and InDels) among 4 experiments. In synthetic control samples processed in all four library preparation experiments (three approaches) the 19065T > C and 26144G > T variants at genomic location were common.

Surprisingly, only sequencing data from libraries prepared by Paragon protocol did not show the presence of variants caused by low coverage at those sites (see Table 1), despite showing the highest mean coverage in comparison to competitors (see Supplementary Table S1). Moreover, the absence of anticipated SNPs is in sharp contrast to significantly higher overall number of called variants in data from Paragon libraries in comparison to both capture-based protocols (NEB+Twist and Illumina), as indicated by the Venn diagram (Figure 5). Both of these findings (low coverage uniformity and high rate of false positives) are in alignment with previously published results [13,14]. Almost 60% of variants in Paragon are common in all tested samples which is caused by PCR biased and low mapping quality (MQ < 20).

All acquired SARS-CoV-2 genomes were uploaded to NCBI Sequence Read Archive (SRA) under accession numbers SUB7745307, SUB7745376, SUB7738659 and SUB7745293.

## 4. Conclusions

In our study we compared the performance of three commercially available NGS library preparation kits. We pointed out factors which are positively/negatively correlated to the data quality of advanced SARS-CoV-2 genome (variant) analysis. From this point of view the Ct value (reflecting the viral load per sample) was determined to be the key predictor of library preparation success and sample data outcome reflected by suitable coverage (at least 20× on a particular sample), regardless of type of workflow. Samples with the Ct value ≥ 23 showed a lower number of total reads and lower ratio of mapped reads, although also these samples (Ct ≤ 23) can be successfully processed, but require higher sequencing capacity. The samples with higher Ct value resulted in low genome coverage—NEB+Twist workflow and Illumina workflow or in absence of expected library preparation product—Paragon workflow. This empirically evaluated dependence also applies to the capture-based approaches where the plexes composed of samples with similar Ct value show higher uniformity of coverage within the plex compared to plexes prepared without respect to Ct value. According to our results the capture-based approaches required a higher sequencing capacity (higher number of reads) to reach the suitable ≥ 20× coverage and showed lower on-target rate than the amplicon-based approach, but the evenness of coverage was better in both capture-based protocols.

## Figures and Tables

**Table 1:** Comparison of detected variant in synthetic control RNA. In our hands, the Paragon approach did not detect all four variants in generated data by standard reference mapping methods.

Position	Twist1	Coverage	Twist2	Coverage	Illumina	Coverage	Paragon	Coverage
19065	yes	390	yes	36	yes	117	yes	66
22303	yes	381	yes	62	yes	166	no	3
26144	yes	217	yes	40	yes	197	yes	45
29749	yes	573	yes	22	yes	58	no	14

## Supplementary Materials

The following are available online at https://www.mdpi.com/2075-4418/10/10/769/s1, Supplementary Table S1: Metrics of samples processed in NEB+TWIST1,2 (NEB+TWIST1 plex 1–3, NEB+TWIST2 plex 2–3), ILLUMINA (plex 1–8) and PARAGON (no plex) approach.; Supplementary Table S2: MiSeq Sequencing. Reagent kits used for sequencing, number of cycles, loading concentration and PhiX spike-in per each sequencing run. Cluster density should be within 1200–1400 K/mm^2^ in case of MiSeq V3 chemistry; Figure S1: Process scheme of time-consumption for library preparation by NEB+TWIST, Illumina and Paragon approaches (extrapolation to 20 samples).
