# Supplementary material for: Performance of Targeted Library Preparation Solutions for SARS-CoV-2 Whole Genome Analysis
Source: Diagnostics (Basel). 2020 Sep 29;10(10):769. doi: 10.3390/diagnostics10100769 (PMC7601271; doi:10.3390/diagnostics10100769)
Supplement: Supplementary file 1 [file diagnostics-10-00769-s001.zip › Proof Supplementary files MDPI/diagnostics-927215-supplementary final/Figure_S1_Process scheme.pdf]

Supplementary materials Figure S1. Process scheme of time-consumption (in minutes) for library preparation by three approaches NEB+TWIST, Illumina and Paragon (extrapolation to 20 samples). The calculation of time was performed based on the manufacturer´s manual and personal experience with workflow, including also relevant QC. First column reflect the particular steps of the workflow (grey rectangles); 2-4 column (NEB+TWIST, Illumina and Paragon) reflect the particular NGS library preparation steps with presented time consumptions (in minutes) related to particular QC (white rectangles with green border line) and workflow steps (white rectangles with grey border line).

	NEB+TWIST	ILLUMINA	PARAGON
**Number of samples**<br>**Sample quality requirement**<br>**Sample type**	20<br>1-100 ng, RIN 2-7<br>SARS-Cov-2 RNA/DNA	20<br>10 ng cDNA, RIN n.spec.<br>SARS-Cov-2 RNA/DNA	20<br>50-100 ng cDNA, RIN n.spec.<br>SARS-Cov-2 RNA/DNA
**Workflow in minutes**	**hands on/instrument**	**hands on/instrument**	**hands on/instrument**
**QC: dCt of samples (qPCR)**	30/90	30/90	30/90
**Fragmentation**<br>**First Strand Synthesis**<br>**Second Strand Synthesis**<br>**Purification (beads)**	5/3<br>20/40<br>20/60<br>20/-	5/1 sec<br>20/60<br>20/60<br>20/-	-<br>20/95<br><br>20/-
**End Prep of cDNA Library**<br>**Adaptor Ligation**<br>**Tagmentation**<br>**Multiplex PCR reaction**<br>**Purification (beads)**	20/60<br>20/30<br>-/-<br>-/-<br>20/-	-/-<br>-/-<br>10/5<br>-/-<br>20/-	-/-<br>-/-<br>-/-<br>20/65<br>20/-
**PCR Enrich. of Adaptor Ligated DNA**<br>**Amplification of Tagmented DNA**<br>**Digestion Reaction**<br>**Purification (beads)**<br>**Second PCR Reaction**<br>**Purification (beads)**	20/23<br>-/-<br>-/-<br>20/-<br>-<br>-	-/-<br>20/20<br>-/-<br>20/-<br>-<br>-	-/-<br>-/-<br>20/10<br>20/-<br>20/50<br>20/-
**QC: Quantification of pre-enriched libraries** (Qubit, ng/µl)<br><br>**QC: Distribution of fragments of pre-enriched libraries** (Bioanalyzer, bp)	20/20<br><br>40/80	20/20<br><br>40/80	20/20<br><br>40/80
**Plexing, concentrating, resuspending**<br><br>**Hybridization**	40/60<br><br>20/960	40/-<br><br>20/960	
**Capturing of Hybridized Probes**<br>**Post-capture PCR amplification**<br>**Purification (beads)**	20/20<br>30/20<br>20/-	20/45<br>30/20<br>20/-	
**QC: Quantification of post-enriched libraries** (Qubit, ng/µl)<br><br>**QC: Distrib. of fragments of post-enriched libraries** (Bioanalyzer, bp)	5/5<br><br>10/40	5/5<br><br>10/40	
**TOTAL time (h, min)**<br><br>TOTAL time (minus hybridisation)	**6h 42min/26h 54min**<br><br>6h 42min/10h 54min	**6h 6min/23h 24min**<br><br>6h 6min/7h 24min	**4h 12min/6h 30min**<br><br>**-**
