# Supplementary material for: Performance of Targeted Library Preparation Solutions for SARS-CoV-2 Whole Genome Analysis
Source: Diagnostics (Basel). 2020 Sep 29;10(10):769. doi: 10.3390/diagnostics10100769 (PMC7601271; doi:10.3390/diagnostics10100769)
Supplement: Supplementary file 1 [file diagnostics-10-00769-s001.zip › Proof Supplementary files MDPI/diagnostics-927215-supplementary final/Table_S2_MiSeq sequencing.docx]

**Supplementary Table S2.** MiSeq Sequencing. Reagent kits used for sequencing, number of cycles, loading concentration and PhiX spike-in per each sequencing run. Cluster density should be within 1200-1400K/mm^2^ in case of MiSeq V3 chemistry.

| **Library Preparation** | **Version of Sequencing Kit** | **Number of Cycles** | **Loading Concentratio*n* (pM)** | **PhiX Spike-In (%)** | **Total Reads (M)** | **PF Reads (M)** | **Yield (Gb)** | **Cluster Density (K/mm^2^)** |
| --- | --- | --- | --- | --- | --- | --- | --- | --- |
| NEB+TWIST1 | v3 (600 cycles) | 2×301 | 17,5 | 5 | 89,97 | 69,88 | 21,39 | 1935 |
| NEB+TWIST2 | v2 (500 cycles) | 2×251 | 9,5 | 1 | 47,06 | 43,8 | 11,23 | 1231 |
| PARAGON | v3 (600 cycles) | 2×201 | 8 | 5 | 67,44 | 62,6 | 12,97 | 1387 |
| ILLUMINA | v3 (600 cycles) | 2×176 | 17,5 | 1 | 79,79 | 71,4 | 13,14 | 1663 |
